# Selective Inhibition of DNA Polymerase Proofreading: A Metabolic–Fidelity Mechanism Explains Agent Orange-Associated Myelodysplasia

**DOI:** 10.3390/cancers18172734

**Published:** 2026-08-23

**Authors:** John J. Byrnes

**Affiliations:** Department of Medicine, Division of Hematology, Sylvester Comprehensive Cancer Center, Miller School of Medicine, University of Miami, 1475 NW 12th Ave, Miami, FL 33136, USA; j.byrnes@miami.edu

**Keywords:** environmental carcinogenesis, myelodysplastic syndrome (MDS), Agent Orange, dioxin, AMP, energy metabolism regulation, mitochondrial dysfunction, DNA polymerase proofreading, exonuclease inhibition, mutation

## Abstract

We performed a focused review to better understand the pathogenesis of Agent Orange (AO)-associated myelodysplastic syndrome (MDS). We first examined the mechanisms underlying conventional (de novo) MDS, a clonal hematopoietic neoplasm that typically develops in later life, and integrated these findings with our recent analysis of obesity-associated carcinogenesis. Accordingly, we propose that genomic instability in de novo MDS results from selective inhibition of the DNA polymerase proofreading exonuclease. In obesity-associated carcinogenesis, impaired AMP-activated protein kinase (AMPK) activity disrupts mitochondrial ATP production, increasing intracellular AMP concentrations. Elevated AMP selectively inhibits the proofreading exonuclease while preserving polymerase activity, allowing replication errors to escape correction and become fixed as somatic mutations. Molecular studies demonstrate that AO-associated MDS exhibits essentially the same mutational profile as de novo disease despite arising after 2,3,7,8-tetrachlorodibenzo-p-dioxin (TCDD) exposure in young, otherwise healthy military personnel. Because TCDD is highly lipophilic, it accumulates in adipose tissue and is released slowly over decades, producing sustained mitochondrial dysfunction, reduced ATP synthesis, and chronic elevation of intracellular AMP. We propose that this metabolic disturbance converges on the same endpoint—selective inhibition of the proofreading exonuclease—thereby promoting mutagenesis and clonal evolution. Recent studies further strengthen the central role of proofreading by demonstrating that many mutations, including many found in MDS, previously attributed to spontaneous cytosine deamination, instead arise from DNA polymerase misincorporation of thymidine opposite cytosine, particularly at CpG dinucleotides, emphasizing the critical importance of fully active proofreading in preventing such misincorporations from accumulating as mutations in the genome of the cell.

## Simple Summary

We reviewed how Agent Orange exposure and obesity may contribute to myelodysplastic syndrome (MDS), a cancer of the blood-forming cells in bone marrow. Although these conditions have different causes, they may damage cells through a shared mechanism. Both obesity and the dioxin in Agent Orange can impair mitochondria, which produce most of the cell’s energy. This lowers ATP, the cell’s main energy source, and increases AMP, a marker of energy stress. We propose that excess AMP interferes with the proofreading function of DNA polymerase, the enzyme that copies DNA when cells divide. DNA copying continues, but errors are less efficiently corrected and can become permanent cancer-promoting mutations. Dioxin may produce long-lasting effects because it accumulates in body fat and is released slowly over many years. This mechanism could explain why Agent Orange-associated and conventional MDS have similar mutation patterns and highlights the importance of DNA proofreading in preventing cancer.

## 1. Introduction

Myelodysplastic syndromes (MDSs) are a group of bone marrow disorders where immature blood cells fail to mature into healthy, functioning blood cells. This leaves the body with low blood counts, which can result in severe anemia or low red blood cells, frequent infections due to low white blood cells, and bleeding problems due to low platelets.

### 1.1. Myelodysplastic Syndromes Are a Malignant Clonal Disorder

For many years, MDSs were regarded primarily as preleukemic disorders because of their propensity to progress to Acute Myeloid Leukemia (AML). Subsequent molecular and clinical investigations demonstrated that MDS itself is a clonal neoplastic disease arising from genetically altered hematopoietic stem cells. Reflecting this evolving understanding, the World Health Organization (WHO) revised the behavior code for MDS in the International Classification of Diseases for Oncology (ICD-O) from 1 (“uncertain whether benign or malignant”) to 3 (“malignant”) in 2000 as a hematological malignancy. Following this reclassification, MDS became reportable to population-based cancer registries, including the Surveillance, Epidemiology, and End Results (SEER) Program, enabling more accurate assessment of its incidence and outcomes.

### 1.2. Obesity and the Risk of MDS

Evidence supporting a relationship between metabolic dysfunction and MDS emerged from epidemiologic studies demonstrating an association between obesity and subsequent development of the disease. In 2009 a significant association between body mass index (BMI) and MDS risk was first reported. Compared with individuals of normal weight, overweight individuals exhibited a relative risk of 1.15 (95% CI, 0.81–1.64), whereas obese individuals demonstrated a markedly increased relative risk of 2.18 (95% CI, 1.51–3.17), indicating a strong dose-dependent relationship between excess adiposity and MDS development [1].

MDSs are now recognized as clonal hematopoietic disorders characterized by ineffective hematopoiesis, peripheral blood cytopenia, dysplastic marrow morphology, and a variable risk of progression to AML. MDS develops through the stepwise accumulation of somatic mutations within hematopoietic stem and progenitor cells, often progressing through recognizable precursor states including clonal hematopoiesis of indeterminate potential (CHIP) and clonal cytopenia of undetermined significance (CCUS) before the emergence of overt dysplasia and leukemia.

### 1.3. Two Types of MDS

Although most cases arise without an identifiable antecedent exposure (“de novo MDS”), a variety of toxic environmental and therapeutic agents have been implicated in disease MDS pathogenesis. Among these, AO, a phenoxy herbicide mixture used extensively by the United States military during the Vietnam War, has received increasing attention. During its manufacture, AO became contaminated with TCDD, one of the most toxic and biologically persistent environmental pollutants known. Exposure to TCDD has been associated with an increased risk of several malignancies, including prostate cancer, soft-tissue sarcoma, multiple myeloma, and non-Hodgkin lymphoma. More recently, evidence has emerged suggesting a relationship between AO exposure and the development of clonal hematopoietic disorders, including MDS.

Support for this association comes from the National MDS Natural History Study, a large prospective cohort conducted across 161 sites in the United States. AO exposure was reported by 6.1% of study participants. Compared with unexposed individuals, exposed patients were more likely to belong to the 1945–1954 birth cohort, reflecting military service during the Vietnam era, and were more likely to receive a diagnosis of either MDS or CCUS (54% versus 37%; *p* = 0.022). After adjustment for differences in sex, race, and military service, AO exposure remained independently associated with a greater number of molecular mutations (OR 1.10; 95% CI, 1.0–1.2; *p* = 0.047), younger age at diagnosis (OR 0.96; 95% CI, 0.93–0.99; *p* = 0.008), and a significantly greater likelihood of poor or very poor cytogenetic risk according to the Revised International Prognostic Scoring System (OR 2.39; 95% CI, 1.04–5.14; *p* = 0.03). Although overall survival did not differ significantly between exposed and unexposed patients (hazard ratio 1.1; 95% CI, 0.7–1.6; *p* = 0.72), AO-exposed individuals demonstrated a substantially higher risk of disease progression, including progression from CCUS to MDS, from lower-risk to higher-risk MDS, or transformation to AML, during the first two years after diagnosis (hazard ratio 1.8; 95% CI, 1.2–2.8; *p* = 0.004) [2]. These observations suggest that AO exposure is associated not merely with the occurrence of MDS but with biologically more aggressive disease characterized by increased genomic instability, adverse cytogenetic features, and accelerated disease evolution. The mechanistic basis for these findings remains incompletely understood.

As MDS has been found to be obesity-associated and a malignant disorder, we propose to analyze both forms of MDS in the context of our recent report on obesity-associated carcinogenesis, and the role impaired energy metabolism and selective inhibition of the DNA proofreading exonuclease as the basis for the associated mutational carcinogenesis [3]. Consequently, the central premise of this review is that the mutation rate of DNA replication may be dynamically regulated by cellular energy metabolism. To accomplish this, a focused review and comparative analysis of the two forms of MDS was performed integrating current and evolving knowledge of their energy metabolism and related mutational consequences within this context.

## 2. Results

### 2.1. MDS Molecular Mutational Profile

To determine whether MDS arising in AO-exposed individuals possesses a distinctive molecular profile, Sperling et al. performed next-generation exome sequencing on bone marrow samples from 29 veterans with AO-associated MDS. As an unexposed comparator, exome sequences from nine patients with MDS treated at the same institution, without known military service or suspected AO exposure, were analyzed and compared with a larger reference cohort of more than 3000 patients with MDS who had undergone molecular profiling using a 95-gene sequencing platform, and genomic alignment and variant calling were conducted using previously validated methods [4].

Analysis identified 55 driver mutations among the 29 AO-exposed patients (range, 0–5 mutations per patient). The majority involved genes commonly implicated in MDS pathogenesis, particularly those regulating RNA splicing (SF3B1, U2AF1, and SRSF2) and epigenetic modification (TET2, DNMT3A, and ASXL1). Importantly, the distribution of driver mutations in the AO cohort closely mirrored that observed in both the institutional control group and previously published sequencing studies of de novo MDS. Thus, the mutational landscape of AO-associated MDS appears fundamentally similar to that of conventional MDS [4].

The predominant mutational events consisted of single-nucleotide variants; primarily, C → T transition mutations occurring at CpG dinucleotides correspond to the well-described aging-associated mutational signature observed in MDS and AML. No significant differences were detected between AO-exposed patients and controls with respect to mutational signatures or overall mutation burden. These mutations were thought to be due to spontaneous cytosine deamination [5].

In summary, the central observation of this study was that MDS arising in AO-exposed individuals exhibits a mutational spectrum remarkably similar to that of de novo MDS in elderly patients rather than a unique molecular signature. Both the driver mutation profile and the underlying mutational processes in AO-associated MDS closely resemble those seen in conventional MDS. These findings argue against AO producing a biologically distinct disease entity and instead suggest that AO exposure promotes the same fundamental clonal and mutational pathways that underlie de novo MDS.

While the mutational landscape of MDSs has been extensively characterized, the factors that govern the mechanism and rate at which mutations accumulate and drive clonal evolution remain incompletely understood. To explore this aspect, it is necessary to examine the mechanisms by which genomic integrity is normally maintained during DNA replication and the pathways that prevent the accumulation of replication errors.

### 2.2. DNA Replication and Mutation Prevention

#### 2.2.1. DNA Replication and Fidelity in Eukaryotes

First, it is necessary to review the essentials of DNA replication fidelity and mechanisms of mutation prevention. Eukaryotic genomic DNA replication is primarily carried out by DNA polymerase δ (pol δ) and DNA polymerase ε (pol ε). Both enzymes possess intrinsic 3′ → 5′ exonuclease proofreading activity, which enables the immediate recognition and removal of misincorporated nucleotides, allowing DNA polymerase to try again to insert the correct nucleotide, and thereby increases replication fidelity by preventing the propagation of misincorporation errors as mutations.

The first eukaryotic DNA polymerase with proofreading activity was identified and purified from mammalian bone marrow in 1976 and designated pol δ [6]. DNA polymerase was recognized as the founding member of a previously uncharacterized class of eukaryotic DNA polymerases. The 3′ → 5′ exonuclease function was determined to reside within its 122-kDa catalytic subunit [7]. In 1980, a second proofreading-competent eukaryotic DNA polymerase was described [8]. Initially considered related to pol δ, this enzyme was subsequently shown to interact with proliferating cell nuclear antigen (PCNA) [9], a sliding-clamp protein essential for highly processive DNA synthesis across extended genomic regions [10].

Further biochemical studies revealed two functional forms of pol δ: a PCNA-dependent and a PCNA-independent form. In 1989, a highly processive DNA polymerase with 3′ → 5′ exonuclease activity and independence from PCNA was purified and characterized. This enzyme contains a 122-kDa catalytic core similar to that of the originally described delta [11]; however it was designated pol ε [12]. As nomenclature evolved, PCNA-dependent polymerases were classified as pol δ, whereas PCNA-independent polymerases were reclassified as pol ε [13]. This reorganization subsequently created terminological confusion: the originally described pol δ, along with the PCNA-dependent form of pol δ, were concurrently isolated from bone marrow and demonstrated to be structurally and immunologically distinct. Importantly, the originally described enzyme was confirmed to be highly processive and PCNA-independent; nevertheless, in accordance with the revised nomenclature, it was retroactively reassigned as pol ε [14], an important distinction for subsequent discussion. Unfortunately, this has led to confusion in subsequent literature.

Both pol δ and pol ε were cloned in the early 1990s, establishing them as the principal replicative polymerases responsible for the bulk of nuclear DNA synthesis in eukaryotic cells [15,16]. Various models have pol ε primarily synthesizing the leading strand in a continuous manner and pol δ the lagging strand in a discontinuous manner. However. this is not settled, and various combinations are also described.

#### 2.2.2. DNA Polymerase Replication Fidelity and Stepwise Improvement

During each cell replication cycle, a human diploid cell genome of 6.4 billion base pairs, or 1.28 × 10^10^ nucleotides, must be copied. The high intrinsic fidelity of the DNA polymerases results in an estimated error rate of 1 in ~10^5^ nucleotide incorporations. However, this error rate would result in roughly ~120,000 base misincorporations when copying the 1.28 × 10^10^ base.

**Figure 1:**
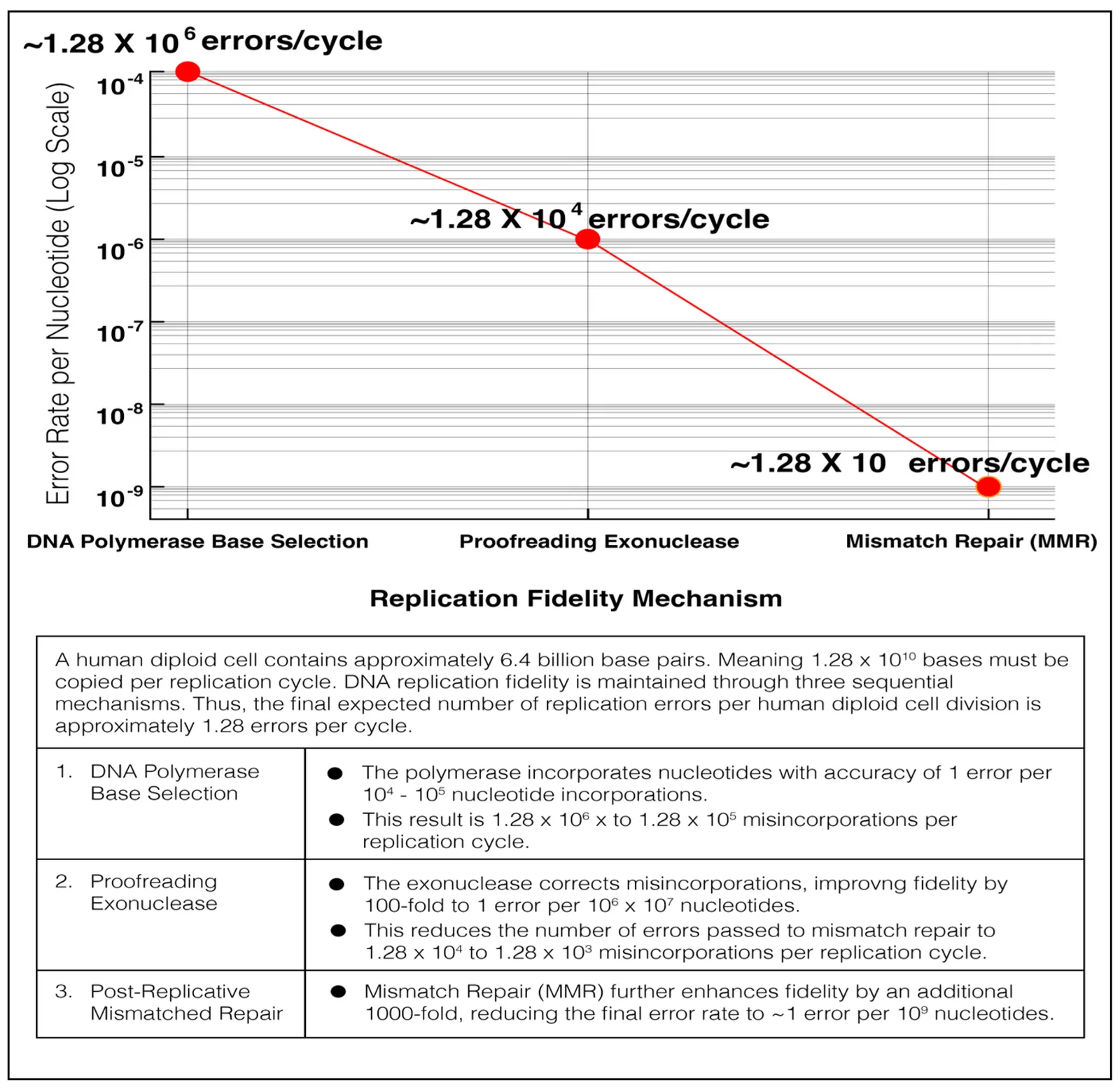
Stepwise reduction of DNA replication errors. Adapted from Byrnes 2026 Figure 2 [3].

The proofreading exonuclease activity of replicative DNA polymerases δ and ε corrects most of these errors, improving fidelity more than 100-fold. The residual errors, about ~1280 mutations, remain, and are distributed between the two daughter cells, yielding ~600 mutations per genome. Subsequently, the post-replicative mismatch repair (MMR) system further corrects remaining mismatches by ~1000-fold, ultimately leaving ~one uncorrected replication error per cell genome (Figure 1) [17].

#### 2.2.3. Energy Requirements for High-Fidelity DNA Synthesis

**Figure 2:**
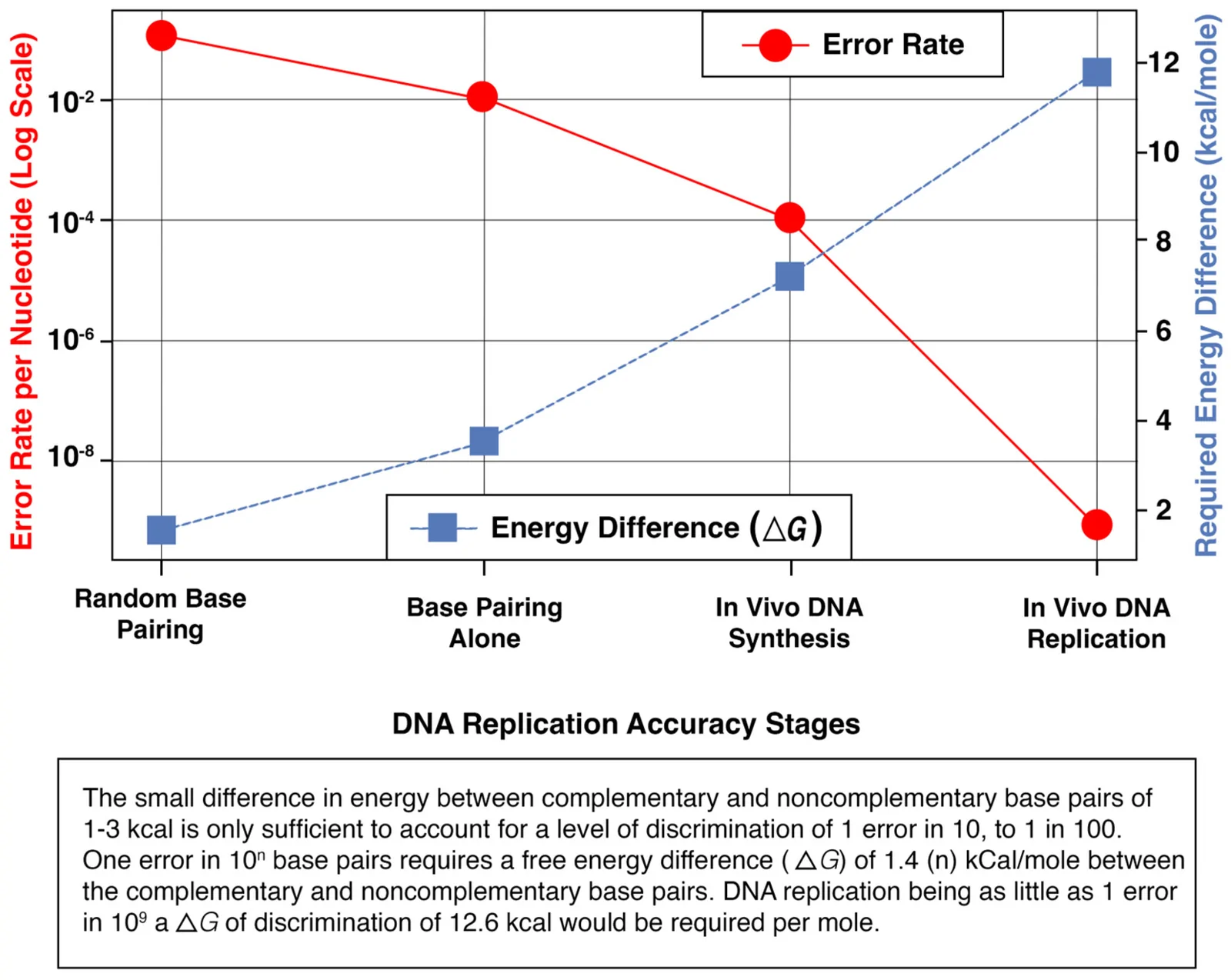
Error rate vs. required energy difference in DNA replication. Figure 2 is a graphical representation of the equation [3] “One error in 10^n^ base pairs requires a free energy difference of 1.4 (n) kcal/mol” e.g., “one error in 10^9^ would require 1.4 × 9 = 12.6 kcal/mol” [18].

Energy is required for DNA synthesis, not only for the substrates and the enzymatic machinery; there is a relationship with a high degree of fidelity and energy that is required (Figure 2). The higher the fidelity, the progressively more energy is required for each error that is corrected.

Proofreading is much more energy efficient than MMR. Proofreading by the replicative DNA polymerase is an intrinsic component of DNA synthesis and requires little additional energy. When an incorrect nucleotide is incorporated, the polymerase’s 3′ → 5′ exonuclease immediately excises the mismatch, and synthesis resumes after insertion of the correct nucleotide. Beyond the energy required to replace the misincorporated nucleotide, the energetic cost is minimal. In contrast, post-replicative mismatch repair (MMR) is a substantially more energy-intensive process. After DNA replication, specialized repair proteins must recognize the mismatch, excise a segment of the newly synthesized strand, resynthesize the deleted DNA, and restore strand continuity by ligation. These sequential steps require repeated ATP-dependent conformational changes and extensive DNA resynthesis, making MMR far more energetically demanding than polymerase proofreading. Consequently, proofreading is energetically efficient for many misincorporations, and MMR is effective at higher fidelity but limited in the number of mutations which can be corrected.

### 2.3. Energy Production and Consumption

#### Energy Regulation by AMP

Adenosine triphosphate (ATP) serves as the primary energy currency in cells, driving a wide array of metabolic processes, including high-fidelity DNA replication. The production and consumption of energy is tightly regulated and coordinated to maintain balance within the cell. Understanding its production and consumption regulation is highly relevant to DNA replication and fidelity maintenance mechanisms.

Daniel Atkinson proposed that adenine nucleotides regulate branch points between anabolism and catabolism based on observations that energy-producing metabolic enzymes that directly monitor the cellular energy state, such as muscle phosphorylase, fructose bisphosphatase, and phosphofructokinase, which were allosterically regulated by adenine nucleotides, with AMP and ATP acting in a reciprocal fashion. This concept was formulated as the Energy Charge Hypothesis [19,20], wherein the energy charge was defined as:

$$\mathrm{energy}\;\mathrm{charge}\;=\;\frac{\left(ATP\right)+\frac{1}{2}(ADP)}{\left(ATP\right)+\left(ADP\right)+(AMP)}$$

Hans Krebs pointed out that the cellular AMP concentrations fluctuate to a much greater extent than those of ATP or ADP. Normally, cells have a much higher concentration of ATP (5–10 mM) than of AMP (<0.1 mM). For example, if ATP is consumed such that its concentration drops by 10%, from 5 mM to 4.5 mM, the AMP concentration that was previously 0.1 mM would increase to approximately 0.6 mM, a 600% increase. Although ATP is the primary metabolite that transports high-energy phosphates during metabolic processes via transitioning to ADP or AMP, Krebs concluded that “the absolute concentration of AMP is a much more sensitive controlling agent than the absolute concentration of ATP” [21]. This concept was further established by the discovery of AMPK which controls cell energy production (ATP) based upon the AMP concentration—a high AMP level indicating a low energy charge and the need for increased ATP generating metabolism and the conservation of ATP by decreasing energy-consuming metabolic processes [22,23].

### 2.4. DNA Replication Fidelity

#### 2.4.1. AMP Selectively Inhibits the Proofreading Exonuclease

Decreased DNA replication fidelity by selective inhibition of the exonuclease activity of DNA polymerases by nucleotide 5′monophosphates, especially AMP, was described in 1977 as an extension of the description of the first eukaryotic DNA polymerase with proofreading exonuclease activity (pol δ, reclassified as pol ε). The addition of AMP to the DNA polymerase reaction selectively inhibited exonuclease activity and decreased fidelity, but left the polymerase activity fully active [24]. The mechanism of selective inhibition was determined to be caused by AMP binding to the product site of the DNA polymerase exonuclease activity [25]. A reason for the functional disassociation of the exonuclease proofreading activity from the DNA polymerase induced by AMP, a prevalent metabolite, remained curiously unexplained, until recently.

#### 2.4.2. AMP Coordinates Fidelity of DNA Replication with Energy Available

A plausible explanation for the selective dissociation of proofreading exonuclease activity from DNA polymerase function by AMP, a ubiquitous cellular metabolite, remained elusive for many years. The apparent paradox was that a common intermediary metabolite could selectively impair replication fidelity while leaving DNA synthesis largely intact. Recent insights into cellular energy regulation suggest that this phenomenon may represent an adaptive response to energetic stress. Under conditions of ATP depletion and AMP accumulation, selective inhibition of the energy-consuming proofreading exonuclease conserves metabolic resources, permitting continued DNA replication at the expense of genomic accuracy. While advantageous for short-term cellular survival, this trade-off allows replication errors to escape correction, thereby allowing mutation accumulation.

#### 2.4.3. Selective Inhibition of the Proofreading Explains Obesity-Associated Carcinogenesis

Obesity-associated carcinogenesis provided a useful model linking metabolic dysregulation to genomic instability and mutation-driven cancer evolution. AMPK coordinates ATP production with metabolic demand; however, in obesity, AMPK activity is impaired, resulting in cellular energy stress characterized by reduced ATP availability and corresponding elevations in AMP.

As AMP concentrations increase, AMP directly binds to and inhibits the 3′ → 5′ exonuclease proofreading activity of DNA polymerases ε and δ. This selective inhibition conserves energy while permitting DNA synthesis and cell replication to continue. The consequence is a reduction in replication fidelity, allowing misincorporated nucleotides to escape correction and become fixed as mutations. Over time, the accumulation of these mutations provides the substrate for clonal selection and carcinogenic evolution.

This model also provides a mechanistic explanation for the well-established observation that obesity increases cancer risk. Therapeutic and lifestyle interventions that activate AMPK—including weight reduction, physical activity, metformin, and aspirin—improve cellular energy balance, increase ATP production, lower AMP levels, and relieve inhibition of exonuclease proofreading. By restoring replication fidelity, these interventions slow mutation accumulation and thereby reduce carcinogenic risk [3].

Consequently, the metabolic mechanism recently proposed for obesity-associated mutational carcinogenesis would be applicable to de novo MDS. In this model, impaired AMPK activity results in reduced ATP production, elevated AMP concentrations, and cellular energy stress, primarily due to the prevalent contemporary lifestyle of obesity and inactivity over many years. Elevated AMP selectively inhibits the proofreading exonuclease activities of DNA polymerases ε and δ, reducing replication fidelity while permitting continued DNA synthesis. Replication errors that would normally be corrected become fixed as mutations, progressively accumulating within hematopoietic stem and progenitor cells. Over time, these mutations confer clonal advantage, leading to clonal hematopoiesis, myelodysplasia, and eventual leukemic transformation. Conversely, interventions that improve cellular energy homeostasis by restoring AMPK activity would be predicted to lower AMP concentrations, preserve proofreading activity, reduce mutation accumulation, and potentially slow disease evolution [3].

What initially appeared to be a biochemical curiosity, the selective inhibition of proofreading by AMP, likely in fact represents a fundamental mechanism linking cellular energy status to mutation acquisition and carcinogenesis. This applies to the development of de novo MDS. However, this mechanistic sequence of impaired AMPK activity in obesity results in reduced ATP production does not apply to AO-associated MDS. In contrast, AO-associated MDS is primarily consequent to a highly toxic exposure in young healthy active men in the military, rather than the prevalent contemporary lifestyle of obesity and inactivity. The metabolic consequences of AO exposure were investigated for possible metabolic connection to the mutational process and mutational signature generation.

### 2.5. Environmental Toxicants and Metabolic Dysfunction

#### 2.5.1. Impaired Glucose Homeostasis

Exposure to dioxins, particularly TCDD, has been associated with metabolic disease phenotypes resembling type 2 diabetes mellitus (T2DM). Epidemiologic studies of herbicide-exposed populations, including veterans of Operation Ranch Hand, demonstrate an increased incidence of diabetes, although authoritative reviews classify the strength of this association as limited or suggestive rather than definitive [26,27,28,29]. Broader analyses of persistent organic pollutants similarly support a relationship between chronic exposure and impaired glucose homeostasis [30,31,32,33].

Mechanistically, dioxin exposure produces a convergent metabolic phenotype characterized by insulin resistance, β-cell dysfunction, hepatic steatosis, and systemic inflammation. Experimental studies demonstrate that activation of the aryl hydrocarbon receptor (AhR) alters pancreatic β-cell function and reduces insulin secretion. Importantly, high-dose TCDD exposure impaired glucose homeostasis and β-cell function in βAhr wild-type mice, but these phenotypes were largely abolished in TCDD-exposed βAhr knockout mice, demonstrating that AhR signaling in β-cells largely mediates the effects of TCDD on glucose homeostasis in both female and male mice, most likely by promoting β-cell inflammation and dysfunction [34] while impairing insulin-stimulated glucose uptake in skeletal muscle.

Im, et al. demonstrated that TCDD induces insulin resistance in muscle cells by significantly impairing insulin-stimulated glucose uptake and GLUT4 translocation. This toxic effect was strongly associated with mitochondrial dysfunction and increased reactive oxygen species (ROS) production [35].

#### 2.5.2. Mitochondrial Toxicity and Impaired ATP Generation

Importantly, Shertzer and colleagues demonstrated that the major toxic contaminant of AO, TCDD, disrupts mitochondrial energy metabolism through direct effects on oxidative phosphorylation. Their studies showed that TCDD exposure decreased intracellular ATP production while simultaneously increasing reactive oxygen species generation. Mechanistically, these changes were associated with alterations in mitochondrial F0F1-ATP synthase and ubiquinone-dependent electron transport processes, indicating impaired efficiency of mitochondrial ATP generation. Thus, TCDD did not merely induce nonspecific oxidative stress, but produced a functional defect in cellular bioenergetics characterized by reduced energy availability and mitochondrial dysfunction [36].

Likewise, Forgacs and colleagues examined the effects of TCDD on the expression of nuclear-encoded mitochondrial genes and demonstrated that TCDD exposure disrupts transcriptional programs essential for normal mitochondrial function. Rather than acting solely as a direct toxicant, TCDD altered the expression of multiple genes involved in oxidative phosphorylation, electron transport, mitochondrial biogenesis, and cellular energy metabolism. Because most mitochondrial proteins are encoded by nuclear DNA and subsequently transported into mitochondria, disruption of these transcriptional pathways has the potential to impair mitochondrial efficiency globally. The findings suggest that TCDD induces a coordinated metabolic reprogramming characterized by reduced mitochondrial functional capacity and impaired energy production. Downregulation of genes involved in oxidative phosphorylation predicts diminished ATP generation with accompanying disturbances in cellular energy homeostasis [37].

Also, Chen et al. examined the relationship between mitochondrial function and dioxin-induced toxicity in JAR cells, a human trophoblast-like cell line. Experiments were performed to address the effects of TCDD on cell viability, reactive oxygen species (ROS) generation, oxidative damage (indicated by the presence of lipoperoxides and oxidized DNA bases), mitochondrial DNA (mtDNA) copy number, ATP content, mtDNA mutations, and the protein levels of p53, Bax, Bcl2, cytochrome *c*, and caspase 3.

Increased oxidative damage and mitochondrial dysfunction in TCDD-treated trophoblast-like cells was demonstrated. A 2.58-fold increase in lipid peroxides was detected in cells treated with 2 nM TCDD for 4 h. The oxidative DNA damage marker 8-hydroxy-2′-deoxyguanosine was significantly increased by TCDD treatment in a time-dependent manner. Meanwhile, reductions in mtDNA copy number and ATP content and an increase in mtDNA deletions were found. Furthermore, increased apoptosis, p53 accumulation, Bax overexpression, cytochrome *c* release, and sequential caspase 3 activation after TCDD exposure were observed. These results indicate that oxidative damage and mitochondrial dysfunction may be responsible for the effects of TCDD [38].

Hwang et al. further strengthened the evidence that mitochondria are primary targets of TCDD toxicity by demonstrating that a population of the AhR localizes within mitochondria, where TCDD directly alters mitochondrial respiration and remodels the mitochondrial proteome. Quantitative proteomic analysis revealed widespread changes in proteins involved in oxidative phosphorylation, electron transport, ATP synthesis, and intermediary metabolism, accompanied by impaired mitochondrial respiratory function. These findings indicate that TCDD disrupts cellular energetics at its principal source by impairing oxidative phosphorylation and ATP generation rather than acting solely through oxidative stress or altered nuclear gene transcription. Such chronic mitochondrial dysfunction provides a biologically plausible mechanism for sustained ATP depletion and energetic stress following AO exposure [39].

A review by Fracchiolla et al. is particularly relevant to understanding the development of MDS following TCDD exposure. TCDD exerts its biological effects through activation of the AhR, a ligand-activated transcription factor that is expressed throughout the hematopoietic system. Thus, TCDD directly reaches and influences the cellular compartment from which MDS arises. Because TCDD is highly lipophilic, it accumulates in adipose tissue and is released only slowly over time, allowing its biological effects to persist for decades. This prolonged persistence is especially relevant to Vietnam veterans, many of whom develop MDS years or even decades after their original exposure.

The review also describes that chronic AhR activation disrupts normal hematopoietic stem-cell function, altering lineage commitment, impairing immune-cell development, and creating conditions favorable for clonal expansion. Hematopoietic differentiation becomes dysregulated, long-term homeostasis is disturbed, and multiple stress-response pathways are activated. These effects are accompanied by metabolic abnormalities that may compromise cellular energetics, as well as aberrant cytokine production, impaired lymphocyte maturation, and chronic inflammatory signaling. These alterations establish a biological environment conducive to the emergence and persistence of abnormal hematopoietic clones, further linking TCDD exposure to the later development of MDS [40].

Cong et al. further demonstrated that TCDD induces intracellular reactive oxygen species, activates AMPK, and disrupts hepatic lipid metabolism through a ROS/AMPK/CD36 signaling pathway. Although ATP and AMP concentrations were not directly measured, AMPK activation is consistent with significant cellular energetic stress resulting from mitochondrial dysfunction. These findings reinforce the concept that TCDD acts primarily as a metabolic toxicant, producing sustained disturbances in cellular bioenergetics rather than functioning solely as a direct genotoxic agent [41].

Sharma and Filippi describe hematopoietic stem-cell aging as a progressive loss of metabolic and mitochondrial control rather than a uniform reduction in oxidative phosphorylation. Quiescent HSCs normally maintain low energy demand, restricted mitochondrial respiration, low ROS, and active mitochondrial quality control. During stress hematopoiesis, however, HSCs exit quiescence and sharply increase glycolysis, mitochondrial membrane potential, oxidative phosphorylation, and biosynthetic metabolism to meet the ATP and substrate requirements of proliferation. Aging impairs this adaptive transition through defective autophagy and mitophagy, lysosomal dysfunction, altered NAD^+^–sirtuin signaling, mitochondrial fragmentation, increased ROS, and chronic inflammatory niche signaling. Consequently, mitochondrial ATP generating capacity may become poorly matched to the increased energy requirements of DNA replication [42].

Collectively, these findings indicate that TCDD exposure produces more than nonspecific oxidative injury; rather, it induces a state of impaired cellular bioenergetics characterized by increased reactive oxygen species (ROS) generation, mitochondrial dysfunction, including reduced oxidative phosphorylation, and decreased ATP production with diminished energy availability. Within this context, the adenylate energy charge concept originally proposed by Atkinson and expanded by Krebs provides a quantitative framework linking impaired metabolism to downstream biological consequences. Small reductions in ATP concentrations are accompanied by disproportionately larger increases in AMP concentrations, making AMP a highly sensitive indicator of cellular energy deficiency. Thus, TCDD-induced impairment of energy metabolism would be predicted to alter cellular energy charge and produce significant elevations in intracellular AMP.

The convergence of environmental toxicant exposure, metabolic dysfunction, and a similar mutational profile suggests the presence of a shared mechanistic axis linking de novo MDS and AO-associated MDS. Although the initiating factors differ, both conditions converge upon a common downstream pathway involving impaired energy metabolism, altered adenylate energy charge, reduced proofreading fidelity, and progressive accumulation of mutations within hematopoietic progenitor populations.

### 2.6. Evidence Further Linking Inhibited Proofreading to the Mutation Spectrum of MDS

#### Evidence Linking Inhibited Proofreading of DNA Polymerase ε to the Mutation Spectrum of MDS

Recent mutational studies clarify the DNA polymerase origin of mutational signatures such as those of de novo MDS and AO-associated MDS and further emphasize the preventive role of the proofreading exonuclease of DNA polymerase ε. As previously mentioned, sequencing studies in AO-exposed veterans and de novo MDS demonstrate that the driver mutations involve *DNMT3A*, *TET2*, *ASXL1,* and spliceosome components such as *SF3B1*, *SRSF2*, and *U2AF1*. Importantly, the dominant nucleotide change observed in these mutated genes is the C → T transition, particularly at CpG dinucleotides. Recent genomic evidence substantially refines the mechanistic framework through which some endogenous mutations arise and accumulate in human cancers, including those involved in both etiologies of MDS. The study by Tomkova et al. [43] demonstrates that one of the most prevalent somatic mutations in cancer, the CpG → TpG transition, arises to a significant extent during DNA replication itself, rather than from the previously given explanation, involving the spontaneous hydrolytic deamination of 5-methylcytosine. Using a polymerase error-mapping approach (PER-seq), the authors show that DNA polymerase ε exhibits a markedly elevated error rate when copying methylated CpG dinucleotides. Furthermore, they showed that cancer-associated exonuclease-domain mutants with impaired function (e.g., P286R) amplify this error spectrum to levels observed in hypermutated tumors. This demonstrates the numerous mutations made by the DNA polymerases in each cell but also the critically important role of the proofreading exonuclease, which is necessary to provide for their correction. This evidence substantially refines the mechanistic framework through which endogenous mutations arise and accumulate in human cancers [43], including those involved in both etiologies of MDS.

### 2.7. Hallmarks of Cancer

In summary and in accordance with the Hallmarks of Cancer paradigm [44,45,46], this model positions impaired energy metabolism as a causal upstream driver of mutational oncogenesis. It explains how metabolic dysfunction both of obesity and of TCDD can precede and promote mutational carcinogenesis.

A central enabling hallmark of cancer is genome instability, which provides the substrate for the acquisition of driver mutations. The present model directly addresses this hallmark by proposing that impaired cellular energy metabolism causing increased cellular AMP reduces the fidelity of DNA replication through selective inhibition of 3′ → 5′ exonuclease proofreading. Extensive experimental and genomic evidence demonstrates that defects in proofreading by the replicative DNA polymerases δ and ε markedly increase mutation rates and are causally linked to carcinogenesis. By introducing a metabolic determinant of proofreading efficiency, this framework extends the concept of genome instability to include both metabolic dysfunction of TCDD as well as that of obesity.

Reprogramming of energy metabolism also is a recognized hallmark of cancer, reflecting the need of proliferating cells to adapt energy production and biosynthetic pathways. In states of impaired energy metabolism—whether induced by environmental toxicants such as TCDD or by systemic conditions such as obesity—reduced ATP production and increased AMP will alter enzymatic processes, especially AMPK, which regulates a diversity of metabolic reactions and would reprogram numerous anabolic and catabolic activities.

Chronic inflammation in obesity is recognized as a key enabling characteristic of cancer. TCDD exposure also induces pro-inflammatory cytokine signaling and oxidative stress, which contribute to both metabolic dysfunction and DNA damage. Within the present framework, DCDD-induced inflammation is an enabling characteristic and acts in parallel with impaired proofreading: reactive oxygen species increase the burden of DNA lesions, while reduced proofreading efficiency increases the likelihood that replication errors persist. The convergence of these processes can synergize and accelerate the accumulation of genomic alterations.

Resisting cell death and cellular stress adaptation is an enabling characteristic of cancer. Cells experiencing metabolic stress must balance survival with maintenance of genomic integrity. The proposed selective inhibition of proofreading under low-energy conditions of obesity or TCDD may represent a trade-off in which cells prioritize survival over fidelity. While this adaptation may confer short-term viability under conditions of metabolic stress, it comes at the cost of increased mutational burden. This concept aligns with emerging views of cancer as a disease of cellular stress adaptation, in which survival pathways are favored even when they compromise genomic stability.

The 2022 expansion of the Hallmarks framework highlights phenotypic plasticity and non-mutational epigenetic reprogramming as key contributors to cancer development. Metabolic dysfunction, including that induced by TCDD exposure, influences epigenetic regulation through changes in metabolite availability and redox state. The present model complements this perspective by introducing a parallel mechanism in which metabolic state influences not only epigenetic regulation but also the fidelity of DNA replication itself. Together, these processes may act synergistically to promote both genetic and non-genetic heterogeneity.

Taken together, this framework positions impaired energy metabolism as a central node linking multiple hallmarks and enabling characteristics of cancer. By influencing both the generation of DNA damage and the fidelity of its synthesis and repair, metabolic dysfunction serves as a determinant of mutational burden. In this context, environmental exposures such as TCDD, like systemic metabolic conditions of obesity, contribute to carcinogenesis not only through proliferative and inflammatory pathways but especially through direct modulation of genome stability by increased cellular AMP, selectively inhibiting the proofreading exonuclease consequent to decreased cell ATP.

## 3. Discussion

These findings shift the conceptual basis of mutational carcinogenesis toward a replication-centered model in which intrinsic, context-dependent polymerase errors constitute a major source of somatic mutations. Within this framework, the role of fidelity mechanisms—particularly 3′ → 5′ exonucleolytic proofreading—must be considered as a central determinant of whether replication-associated errors are corrected or fixed within the genome. The present hypothesis, proposing selective inhibition of the proofreading exonuclease by elevated AMP in states of impaired energy metabolism, integrates directly into this model.

In this context, selective inhibition of the exonuclease function would not create new classes of mutations but would permit the persistence of an already biased spectrum of replication errors. The increased error rate observed at methylated CpG sites identifies a genomic context in which the replication machinery operates near the limits of fidelity. Even modest reductions in proofreading efficiency in such contexts would be expected to disproportionately increase the fixation of CpG → TpG transitions, thereby linking metabolic state to characteristic mutational signatures observed in many human cancers.

This interpretation aligns with established genetic evidence demonstrating that exonuclease-domain mutations in polymerases δ and ε produce hypermutator phenotypes and early-onset tumorigenesis. However, the metabolic model proposed here extends these findings by suggesting a physiological, reversible analogue of the mutator phenotype, mediated not by structural mutation of the polymerase, but by modulation of its proofreading activity through cellular energy charge. In states such as obesity, where impaired AMPK signaling leads to decreased ATP and elevated AMP, the selective binding of AMP to the exonuclease site may inhibit proofreading without impairing polymerase synthesis. This would conserve energy while reducing replication fidelity, thereby increasing the retention of replication-associated errors, similarly to dioxin-impaired ATP synthesis, where direct toxic impairment of cell mitochondrial ATP metabolism is impaired, with consequent increase in cellular AMP.

The implications of this proposed model are twofold. First, it provides a mechanistic basis for the epidemiologic association between metabolic disorders and toxic disorders and cancer incidence, linking impaired energy metabolism to increased mutational burden through a defined biochemical interaction. Second, it predicts that metabolic interventions that restore ATP levels and reduce AMP—such as weight loss, exercise, or pharmacologic activation of AMPK—may decrease mutation accumulation by restoring proofreading efficiency. This introduces a temporal dimension to prevention strategies, in which long-term modulation of replication fidelity influences the rate of somatic mutation acquisition.

Notably, the findings of Tomkova et al. also underscore the importance of sequence context and epigenetic state in determining mutational outcomes. The preferential vulnerability of methylated CpG sites suggests that metabolic modulation of proofreading may not act uniformly across the genome, but rather may amplify specific, context-dependent mutational processes. This provides a potential explanation for the non-random distribution of mutations in cancer genomes and supports the concept that metabolic state may influence not only the quantity but also the qualitative pattern of somatic mutations.

In summary, recent genomic evidence supports a model in which DNA replication is a primary source of common somatic mutations, with the pol ε playing a central role in generating context-dependent errors. The proposed selective inhibition of proofreading exonuclease activity by elevated AMP complements this model by providing a mechanism through which these replication errors may be preferentially retained under conditions of impaired energy metabolism. Together, these findings support a unified framework in which intrinsic polymerase error bias and metabolically regulated proofreading efficiency jointly determine mutational burden and carcinogenic risk. This framework also accommodates the two different clinical scenarios of impaired energy metabolism with decreased ATP, both of which result in the same mutational profile. This further supports the concept of increased AMP modulation of the proofreading exonuclease as a basis of mutational carcinogenesis.

The recent publication of Tomkova et al. provides further critical mechanistic evidence that DNA polymerase ε is a direct source of C → T mutations, a prevalent mutational signature in both aging and carcinogenesis. The similarity between AO-associated and age-related MDS therefore supports a model in which environmental exposure accelerates an endogenous replication-error process. Selective inhibition of the proofreading exonuclease activity by increased AMP offers a biologically plausible mechanism linking metabolic or toxic energy stress to increased mutational burden, clonal evolution, and the development of myelodysplasia.

## 4. Conclusions

This model reframes MDS—both MDS de novo and AO-associated MDS—as a disorder of energy-dependent genomic maintenance, in which the rate of mutation accumulation is governed not only by intrinsic polymerase error rates but also by the metabolic capacity to correct those errors. The proofreading exonuclease thus emerges as a critical energy-regulated gatekeeper of genomic fidelity. Its selective inhibition provides a unifying mechanism linking metabolic dysfunction to mutagenesis, clonal hematopoiesis, and myelodysplasia.

Importantly, this synthesis suggests that interventions which restore energy balance, such as AMPK activation, may enhance replication fidelity by relieving exonuclease inhibition. Such strategies may therefore reduce mutation accumulation and alter the trajectory of MDS development in both aging and environmentally exposed populations. The prevention of exposure to toxins such as TCDD is emphasized by understanding the severity of the mechanism. The necessity of avoidance of toxic exposure is also emphasized as, once mutations are established, the corrective mechanisms are limited.

## 5. Practical Implications

This synthesis encompasses several practical implications. Impaired AMPK in obesity provides a link between disordered energy metabolism and the mutagenesis leading to de novo MDS. This framework supports existing recommendations for weight control, diet quality, and physical activity, potentially improving long-term adherence. Importantly, the predicted reduction in MDS risk operates on an extended time horizon: if MDS is driven by DNA replication errors whose frequency of correction is modulated by cellular energy status, then metabolic correction reduces the rate of mutation accumulation rather than reversing existing damage. Consequently, measurable hematological benefit may not be evident for many years, and the patient may not fully appreciate something that does not occur. In contrast, improvements in cardiometabolic health, including insulin sensitivity, hepatic steatosis, glycemic control, and systemic inflammation, often can occur within weeks to months, and should be emphasized as primary motivators. MDS risk reduction in conjunction with the risk reduction of the other cancers associated with obesity should be framed as a long-term consequence of sustained metabolic normalization.

MDS in association with environmental toxins such as AO can only be prevented by avoidance. We hope the understanding of the molecular mechanism conveys the seriousness of exposure and will motivate measures to provide for avoidance. The measures advocated for prevention of de novo MDS would have some effect in attenuating the consequent trajectory of AO mutation accumulation. That advice also pertains to the risk of the other cancers also associated with AO, as well as those associated with obesity.

We hope this synthesis of cellular energy metabolism and its relationship to the two etiological forms of MDS, particularly in terms of preventive strategies, will encourage its further development. The current paradigm for the medical treatment of MDS is formulated based on the analysis of the specific mutations involved and options available. Although our recommendations are primarily for prevention, they may have application in some of the less advanced clinical presentations in slowing their evolution into leukemia. Clinical trials may be necessary; however, lifestyle modifications may be recommended, adjusting for clinical circumstances.

The scientific and pharmacological discovery of products for AMPK activation is an area of substantial interest, with numerous natural products and several synthetic compounds under consideration for further development. There is intense interest in the pharmacological activation of AMPK as a therapeutic strategy for multiple disease states, including obesity, diabetes, and associated cancer. Pharmacological agents, such as GLP-1 receptor agonists, are now starting to show effectiveness in the prevention of cancer [47,48,49]. Finally, continuous AMPK overstimulation may have adverse tissue-specific consequences, underscoring that prevention aims to restore physiological, context-dependent AMPK regulation rather than induce chronic activation.

### Strengths, Limitations, and Future Directions

A major strength of this framework is that it provides a metabolically and thermodynamically coherent explanation for the mutational evolution of both de novo and AO-associated MDSs. By integrating cellular energy metabolism with the mechanisms governing DNA replication fidelity, it offers a unified model linking impaired bioenergetics and genomic instability. Beyond explaining disease pathogenesis, this framework also identifies potential strategies for prevention and therapeutic intervention through preservation or restoration of cellular energy homeostasis.

Several limitations should be acknowledged. Much of the current evidence regarding the metabolic effects of AO and its contaminant TCDD derives from studies of non-hematopoietic tissues, particularly liver and muscle. Although these findings are biologically relevant, their application to hematopoietic stem cells necessarily requires extrapolation. Direct investigation of energy metabolism and adenylate homeostasis within hematopoietic tissues remains an important priority. Appropriately, studies on the original Pol δ and the associated proofreading exonuclease and its selective inhibition were performed with enzymes obtained, isolated, and purified from mammalian bone marrow, the primary hematopoietic tissue. Future investigations should therefore focus on correlating intracellular adenylate concentrations, mitochondrial function, DNA replication fidelity, and mutational evolution within hematopoietic stem and progenitor cells. Modern genomic technologies—including high-fidelity DNA sequencing, mutational signature analysis, and single-cell approaches—combined with quantitative metabolomic measurements should make it possible to directly test the proposed relationship between cellular energy status and mutation acquisition. Such studies would provide critical validation of this metabolic–fidelity model and may identify new opportunities for the prevention and treatment of MDSs.

## Data Availability

No new data were created or analyzed in this study. Data sharing is not applicable to this article.
